# Associations of Scoring Accuracy with Postural Stability and Strength Measures in Archers on a Standard Archery Site

**DOI:** 10.3390/sports13090310

**Published:** 2025-09-08

**Authors:** Chun-Hao Fan, Chien-Nan Liao, Wei-Hsiu Hsu

**Affiliations:** 1 Office of Students’ Affairs, Yanshuei Elementary School, Tainan 73746, Taiwan; fun711009@gmail.com; 2 Graduate Institute of Education, National Chung Cheng University, Chiayi 621301, Taiwan; 3 Department of Athletic Sports, National Chung Cheng University, Chiayi 621301, Taiwan; liao.cody@gmail.com; 4 Sports Medicine Center, Chang Gung Memorial Hospital at Chiayi, Chiayi 613016, Taiwan; 5 Department of Orthopedic Surgery, Chang Gung Memorial Hospital at Chiayi, Chiayi 613016, Taiwan; 6 School of Medicine, Chang Gung University, Taoyuan 33302, Taiwan

**Keywords:** compound archery, center of pressure, handheld dynamometer, inertial measurement units, exercise training, coaching strategies, recurve archery

## Abstract

Archery performance is substantially influenced by postural stability. Although archery is commonly practiced outdoors, most studies have focused on short-distance indoor environments. Accordingly, this study examined the correlation between postural stability and shooting accuracy in competitive recurve and compound archers on a standard outdoor field (70 m for recurve and 50 m for compound). This study included 37 archers. Each archer’s performance was recorded during a simulated competition. Measurements included muscle strength, body stability, and center of pressure. Postural stability data were analyzed at 0.5 s before and 0.1 s after arrow release. The results indicated that compared with compound archers, recurve archers had stronger upper-limb muscles and exhibited lower pre-release total center of pressure (51.9 mm; *p* = 0.022) and medial/lateral sway (1.1 mm; *p* = 0.043). The compound archers exhibited lower post-release anterior/posterior sway (3.2 mm; *p* = 0.001) and lower angular velocities in most body segments, except for the lower back. The recurve archers relied more on post-release stability, whereas the compound archers relied more on pre-release control. Linear regression analysis identified different predictors of scoring accuracy for each bow type. Our findings highlight the need for discipline-specific training strategies, such as enhancing bow-side stability for recurve archers and drawing-side control for compound archers.

## 1. Introduction

Archery has a long and rich history and was officially introduced to the Olympic Games in 1972. Currently, archery is enjoyed by millions of people of all ages and abilities worldwide, including individuals with physical limitations who participate in adaptive recreational or school physical education programs [1]. Archery performance refers to the ability to accurately shoot an arrow at a designated target. Numerous studies have examined the factors influencing archery performance and have highlighted the characteristics of elite and highly skilled archers: the capacity to consistently hit targets within a specific time frame with a high degree of precision [2,3,4,5]. Studies have also emphasized the importance of physical fitness indicators, such as core muscle strength, upper-body strength, handgrip strength, leg power, and static balance, in archery performance [6,7,8].

Increasing the strength of core muscles and stabilizing the muscles of the spine, scapula, and pectoral girdle substantially contribute to postural control, which is a key determinant in initiating movement and achieving optimal performance in sports [9]. In archery, players are required to maintain maximal postural stability by minimizing their arm and trunk movements while drawing the bow and aiming [9,10].

Archery is a static sport that demands shooting posture standardization and body sway minimization, including control of center of pressure and lower bow sway, during the aiming phase to enhance shot accuracy [3,11]. Maintaining postural stability is essential for achieving consistently high scores in archery, and the limb used to support the bow side during shooting is a key factor influencing body sway control and shot accuracy [12,13]. During the arming phase, rapid activation of fast-twitch muscle fibers is necessary to generate a sufficient pulling force on the bowstring, and during the subsequent sighting phase, archers must maintain their posture with high precision [14]. A regular, smooth, and steady final pull is essential for achieving high archery performance [15]. However, tremors are sometimes observed during the sighting phase, which are believed to be primarily postural and isometric in nature [14].

In recent years, archery has attracted considerable research attention, with relevant studies primarily focusing on aspects such as training strategies [16], talent identification [8], and sports injuries [17]. However, most data on body stability, shoulder joint kinematics, and surface electromyography have been collected in indoor laboratory settings, where shooting distances typically range from 5 to 30 m [5,17,18]. This range is substantially shorter than the standard competition distance, which is usually 50 or 70 m. Clarys et al. [19] reported substantial differences in muscle activation intensity between indoor and outdoor shooting settings, indicating the importance of context in archery performance analysis.

The compound bows are mechanically more complex, with cams and pulleys that assist in drawing the string, reduce holding weight for easier aiming, and provide more power and speed when releasing the arrow. Recurve bows require the archer to physically hold full draw weight, rely more on skill and strength for aiming, and have a simpler release mechanism without mechanical assistance. Currently, many grassroots archery teams train recurve and compound archers together without distinguishing between the training methods for each discipline. Moreover, most studies on archery have primarily focused on recurve archery [4,8,20], with limited studies discussing compound archery. Accordingly, to address this gap, we expanded the scope of our investigation to include comparative analyses between recurve and compound archers. Specifically, we investigated postural stability measures, including whole-body stability and center of pressure (COP), through sports biomechanics analysis. We also examined the correlations between these stability measures and scoring accuracy under standard outdoor competition conditions for recurve archery (70 m) and compound archery (50 m). Here, we hypothesized that recurve and compound archers would exhibit different postural stability and be associated with distinct predictive factors.

## 2. Materials and Methods

### 2.1. Participants

This prospective study recruited archers aged 15–24 years from high school and university archery teams in Taiwan between January and July 2024. All archers had completed a minimum of 3 years of structured and professional archery training. The participants were pre-elite high school and university archers, defined as athletes competing regularly at the national level but without international experience. Scores from ranking rounds closest to the testing date were recorded, averaging 550 (465–638) for high school recurve, 624 (599–657) for university recurve, 663 (639–684) for high school compound, and 670 (644–689) for university compound archers. Performance during a simulated competition was recorded as the primary outcome. Additionally, muscle strength, body stability, and COP were analyzed to explore their associations with archery performance.

All participants provided written informed consent before being enrolled in this study. All of them were right-handed; in other words, they used their right hand during the bow-drawing phase. Archers were excluded if they (1) had a history of surgery to either shoulder, (2) had a shoulder muscle disease, or (3) had shoulder impingement syndrome. This study was approved by the Institutional Review Board of the Chang Gung Medical Foundation (approval number: 202301149B0).

### 2.2. Study Design and Procedure

This study measured the following anthropometric and performance parameters: age, height, body weight, postural stability (limb and trunk stability, COP), arrow scores, and upper-limb muscle strength. Stability and COP were simultaneously recorded during each shot.

All testing procedures were conducted on a standard outdoor archery range (70 m for recurve and 50 m for compound) under controlled weather conditions. Upon arrival, participants completed a standardized 10 min warm-up consisting of dynamic stretching and light shooting to ensure readiness. Each participant then executed six shots toward a standard competition target. After completing the postural stability test, the athletes were given a 30 min rest period before undergoing the strength assessment.

During shooting, postural stability was assessed using a force plate, inertial measurement units (IMUs), and a high-speed camera system, all operated via the Nexus motion analysis system (version 2.5.0; Oxford Metrics, Oxfordshire, UK). To ensure measurement reliability, all devices were calibrated before each data collection session according to the manufacturer’s guidelines. The same examiner team conducted and monitored all trials to maintain consistency. In addition, shooting stance, equipment setup, and trial sequence were standardized across all participants to minimize variability.

The primary outcome of this study was a regression analysis examining the association between postural stability and scoring accuracy.

### 2.3. Arrow Shooting

Environmental factors such as location and weather may influence the competitive ranking or athletic performance of archers [21,22]. Therefore, in this study, all measurements were conducted under the same weather conditions. The temperature was maintained at approximately 26–33 °C, relative humidity at 75–80%, and there was no measurable wind speed. After a regular warm-up session, each participant was asked to shoot six arrows toward a target placed at a distance of either 70 m (recurve archery) or 50 m (compound archery). All shooting attempts were made on traditional five-color target faces with 10 concentric scoring rings. For recurve archery, the target face had a diameter of 122 cm, with the 10-ring having a diameter of 12.2 cm. For compound archery, the target face had a diameter of 80, with the 10-ring having a diameter of 8 cm. A score of 10 points was awarded for hitting the innermost ring, whereas a score of 1 point was awarded for hitting the outermost ring. Scores of 10 and 9 points were awarded for hitting the yellow rings, scores of 8 and 7 points were awarded for hitting the red rings, scores of 6 and 5 points were awarded for hitting the blue rings, scores of 4 and 3 points were awarded for hitting the black rings, and scores of 2 points and 1 point were awarded for hitting the white rings. A score of 0 points was provided for missing the target altogether. Each archer was asked to execute six shots at their own convenience for data collection.

### 2.4. Postural Stability

Archers’ postural stability was assessed using inertial measurement units for whole-body stability and a force plate for COP data [23,24]. Previous studies have shown that the arrow leaves the bowstring within 15–20 ms after release in both recurve and compound bows [25,26]. Related work has also examined EMG activity 0.1–0.5 s before release [27] and COP displacements from 1 s before to 0.5 s after release [4]. Building on these findings, the present study analyzed biomechanical data within the window of 0.5 s before to 0.1 s after each shot.

In this study, all participants used their left foot as the supporting limb during the shooting phase. Kinetic data were collected from the left foot by using an AMTI force plate (ACG-O; AMTI, Watertown, MA, USA) at a sampling rate of 1000 Hz. Relevant studies have consistently demonstrated its good reliability and validity [28,29]. Sway or postural stability was subsequently calculated by measuring the change in COP during each shot cycle [30]. The COP trace was divided into COPx (representing medial/lateral sway, across the target’s face) and COPy (anterior/posterior, in line with the target) [4].

Angular velocity was recorded at 1000 Hz by using inertial measurement units (Blue Trident; Oxford Metrics, Oxford, UK) placed on each participant’s left ankle, lower back (L4 spinous process), left wrist, and right wrist to represent their limb and trunk stability [12,13,31]. Previous studies have confirmed the reliability and validity of sport-specific IMUs, with data automatically processed by the manufacturer’s software to provide a comprehensive and practical biomechanical load monitoring system [32]. A resultant vector was calculated from the corresponding X–Y–Z vectors from each gyroscope. The root mean square was then calculated for each gyroscope, across each orthogonal plane and the resultant vector [33].

The moment the arrow was released was defined as the point at which the fingers on the string (the drawing side) were straightened [16]. Shooting motion was captured using a high-speed camera (Vue; Oxford Metrics, Oxford, UK) at 100 Hz.

**Figure 1:**
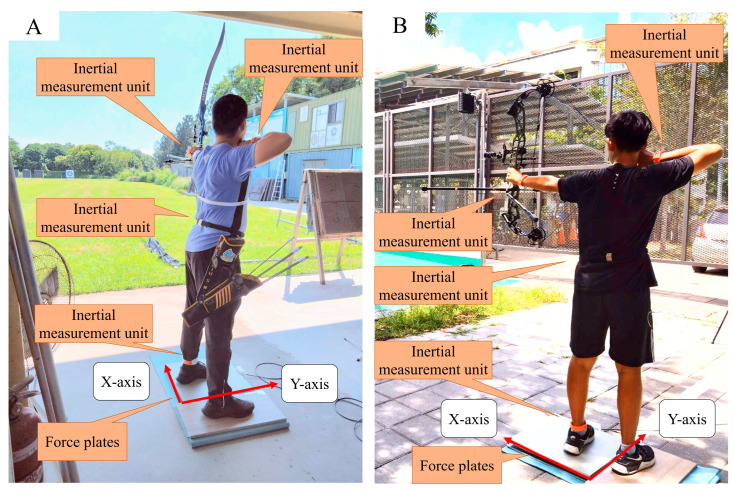
Experimental setup: (**A**) recurve bows; (**B**) compound bows.

The force plate, inertial measurement units, and high-speed camera were synchronized and recorded using the Nexus motion analysis system (version 2.5.0; Oxford Metrics, Oxfordshire, UK). To ensure consistency across trials, all participants underwent a familiarization session before data collection and were instructed to shoot in their usual competition stance, while equipment setup, calibration, and system synchronization were performed prior to each recording session. Figure 1A,B present the experimental setup.

### 2.5. Muscle Strength

All participants were provided with standardized instructions before measurement. Before the strength assessments, all participants completed a standardized 5 min warm-up routine involving dynamic stretching of the shoulder and upper back muscles. The warm-up emphasized mobilization of the scapular and shoulder regions to reduce stiffness and facilitate optimal muscle activation for the subsequent strength testing. For all measurements, the testing sequence was standardized, beginning with the lower trapezius (right side followed by left side), followed by the shoulder horizontal adductor (right then left), the shoulder horizontal abductor (right then left), and finally the upper trapezius (right then left). To measure the strength of the lower trapezius (LT), each participant was asked to assume the prone position with their test arm placed diagonally overhead, aligned with the direction of the LT muscle fibers, as described by Petersen and Wyatt [34]. To measure the strength of the upper trapezius, each participant was asked to sit with their arm elevated, and then a depression force was applied to the superior aspect of the shoulder by using a handheld dynamometer, which was manually stabilized by the examiner [35]. Shoulder horizontal adductor strength was measured in the supine position with the shoulder abducted to 90°, in neutral rotation, and the elbow flexed at 90°, and in the opposite direction when testing shoulder horizontal abductor strength. The test arm was positioned at the edge of the examination table, as described by Hirano and Katoh [36]. The dynamometer’s sensor was placed at the distal one-third of each participant’s radial forearm, and resistance was applied in the downward or upward direction until maximal voluntary effort was overcome. All measurements were performed using a MicroFET 2 handheld dynamometer (Hoggan Health Industries, West Jordan, UT, USA). The handheld dynamometer has demonstrated moderate to excellent test–retest reliability and high concurrent validity [37]. The maximum force on the dynamometer was recorded. Three trials were consecutively conducted on each arm, with an intertrial rest period of 30 s. Finally, the average for each side was used for analysis. All tests were performed by the same examiner to minimize inter-rater variability.

### 2.6. Statistical Analysis

All statistical analyses were conducted using IBM SPSS Statistics for Windows (version 20.0; IBM, Armonk, NY, USA). Continuous variables are expressed as mean ± standard deviation. No abnormal distributions were noticed in pre-tests according to the Shapiro–Wilk results for both groups. The relative effect size (ES) for the performance data was calculated using Cohen’s *d*. It is defined as the difference between two mean values divided by a standard deviation for the data. Categorical variables were compared using the chi-square test. Additionally, the effect size of categorical variables was evaluated using Cohen’s *ω*. Group differences based on bow type were analyzed using independent-samples *t* tests. A *p* value of <0.05 was considered to indicate statistical significance. Pearson’s correlation analysis was used to examine the associations between variables. To identify significant predictors of arrow scores, multiple linear regression analyses were conducted using a forced-entry method. The primary outcome of this study was regression analysis. Based on a significance level of α = 0.05 and statistical power of 0.80, a total of 114 arrows from recurve archers were included in the analysis. The estimated effect size was f^2^ = 0.15, which is classified as a medium effect according to Cohen’s criteria.

## 3. Results

A total of 37 archers were included in this study. These archers were divided into a recurve archers group (*n* = 19) and a compound archers group (*n* = 18). The recurve archers group had a mean age of 19.0 years and a body mass index of 25.9 kg/m^2^, and the compound archers group had a mean age of 18.7 years and a body mass index of 23.9 kg/m^2^. A significant between-group difference was observed in body weight, although the two groups had similar demographic characteristics. Upper-limb strength measurements revealed that the recurve archers group had significantly greater horizontal abductor (19 kgf) and adductor (15.5 kgf) strength on the drawing side and greater LT (6.5 kgf) and horizontal abductor (19.1 kgf) strength on the bow side when compared with the compound archers group. The average score per arrow was 9.1 points in the compound archers group and 8.3 points in the recurve archers group, indicating a significant difference (Table 1).

The total COP displacement at 0.5 s before arrow release was 51.9 mm in the recurve archers group and 61.6 mm in the compound archers group. In terms of medial/lateral sway, the recurve archers group had a sway value of 1.1 mm, whereas the compound archers group had a sway value of 1.5 mm. Regarding anterior/posterior sway, the two groups exhibited sway values ranging from 3.0 to 3.3 mm. At 0.1 s after arrow release, no significant difference was observed between the two groups in terms of total displacement or medial or lateral sway; however, the compound archers group exhibited significantly lower anterior and posterior sway compared with the recurve archers group. At 0.1 s after arrow release, the angular velocities of the left ankle, lower back, bow-side wrist, and drawing side wrist were approximately 2.2–3.9°/s, 19.8–29.3°/s, 104.7–132.6°/s, and 215.5–271.4°/s, respectively. Compared with the recurve archers group, the compound archers group exhibited smaller angular velocities in all other body parts, except for the lower back, in which no significant between-group difference was observed (Table 2).

Both the compound and recurve archers groups exhibited a moderate to weak negative correlation between score per arrow and postural stability, with lower COP displacement and angular velocity being associated with higher accuracy. Additionally, a moderate to strong positive correlation was observed between COP displacement and angular velocity (Supplementary Tables S1 and S2). After strongly correlated variables were excluded and after sex, age, and body weight were adjusted for, linear regression analysis revealed that the total COP displacement after arrow release and the angular velocity on the bow side were predictive of score per arrow in recurve archery, suggesting that reducing COP displacement and shaking on the bow side can improve accuracy (Table 3).

The equation for the scores of recurve archers is as follows:

Scores of recurve archers = 14.502 + (−0.109 × COPd) + (−0.007 × angular velocity on the bow side)

By contrast, for the compound archers group, smaller medial/lateral COP displacement 0.5 s before arrow release and reduced shaking on the drawing side were found to be associated with higher accuracy (Table 4).

The equation for the scores of compound archers is as follows:

Scores of compound archers = 11.847 + (−0.121 × COPy) + (−0.191 × angular velocity on drawing side)

## 4. Discussion

This study revealed that recurve archers exhibited significantly greater upper-limb muscle strength compared with compound archers. This difference is attributable to the mechanical advantage provided by the cam system of compound bows, which reduces the force required to maintain full draw, thereby increasing the stability of the aiming process [38]. Recurve bows have a traditional design that requires maintaining a great drawing force at full draw [15]. Therefore, recurve archers must have great upper-limb muscular strength to effectively maintain their postural stability and ensure precise motor control throughout the shooting process.

Our findings reveal a negative correlation between score per arrow and COP displacement after arrow release. They also reveal a negative correlation between score per arrow and reduced stability in the left foot and bow side. These findings are consistent with those of previous studies [5,14]. Notably, we observed that in recurve archery, bow-side stability 0.5 s before arrow release was negatively correlated with the stability of both hands 0.1 s after arrow release. We also determined that bow-side stability after arrow release was negatively correlated with the COPx and COPy values before arrow release. In the compound archers group, the stability of the drawing side and lower back was negatively correlated with COP displacement both before and after arrow release. These patterns were not observed in studies involving elite recurve archers [4,39,40]. In our study, the average age of the participants was approximately 19 years, indicating that their archery skills and experience were still under development. Quan and Lee [12] reported that the movement directions of specific body segments influencing shot accuracy varied among four novice archers. These findings suggest that developing archers are influenced by diverse and individualized performance factors. Such variability may explain the negative correlations observed between COP displacement and distal limb stability before and after arrow release, potentially reflecting immature muscle control and coordination.

Our results indicate that both COP displacement and bow-side stability at 0.1 s after arrow release were significant predictors of shot accuracy in recurve archers. These results are consistent with those of Quan and Lee [12], who demonstrated that the stability of the bow side and left foot (supporting foot) significantly influenced archery performance. Spratford and Campbell [4] identified postural sway speed after arrow release as a key determinant of scoring performance among elite archers, with lower sway speeds associated with higher shot accuracy. These findings underscore the importance of maintaining postural control immediately after arrow release. After the aiming phase, elastic energy is transferred from the bow to the arrow, resulting in forward bow displacement due to its inherent inertia, a predictable biomechanical response. At this moment, the archer must maintain their postural stability and balance, particularly on the bow side, to counteract changes in COP and body equilibrium induced by the bow’s reactive motion [39]. In addition, Lau et al. [40] argued that the elbow’s deviation angle after arrow release plays a crucial role in determining shot outcomes in recurve archery.

Our regression analysis revealed that the performance-related factors of compound archery differed from those of recurve archery. Specifically, anterior and posterior COP displacement before arrow release and stability on the drawing side were significant predictors of shooting accuracy. Kim et al. [41] also reported that lower bow displacement and tremor during the aiming phase and lower COP displacement and speed were associated with higher accuracy. However, neither bow displacement nor postural sway after arrow release was significantly associated with shooting performance. Wu et al. [18] indicated that the use of arch-support insoles by compound archers during the arrow release phase can effectively reduce foot COP excursion and subsequently enhance shooting performance. These findings are consistent with those of the present study.

Our findings indicate that postural control plays a critical role in determining archers’ shooting accuracy. For recurve archers, archery performance primarily depends on their stability from the moment of arrow release, whereas for compound archers, archery performance mainly depends on their postural stability before arrow release. In recurve archery, the archer must maintain control against constant resistance that begins from the moment of drawing the bowstring until its release. However, in compound archery, this resistance is lower [42]. Therefore, compared with compound bows, recurve bows generate greater reactive forces at the moment of arrow release. Hence, recurve archers typically require high postural control after arrow release to maintain their stability and shooting accuracy. In recurve archery, the muscular effort needed to counteract the bow’s reactive force, in conjunction with the demands of maintaining postural equilibrium, is reflected in quantifiable changes in ground reaction forces and center of pressure displacement between the archer and the ground [39].

From a practical perspective, training strategies should reflect these demands. For recurve archers, incorporating unstable resistance training may help simulate the vibrations experienced after arrow release. Oscillation exercises, a form of such training, have been shown to enhance core and limb muscle activation by engaging stabilizing muscles that support joint integrity [43,44]. Such exercises may improve performance by promoting postural control and muscular coordination. For compound archers, stability can be enhanced through exercises such as side bridges [14], core stability exercises [9], and lower trapezius training [16], which improve pre-release control and shooting precision. More broadly, coaches may consider integrating stability-focused training programs, including balance exercises and individualized upper-limb conditioning, to enhance archers’ ability to maintain control during shooting. Additionally, monitoring biomechanical parameters using tools such as force plates or inertial sensors could provide valuable feedback for both performance optimization and injury prevention. Finally, tailoring training approaches to the specific demands of recurve and compound archery may enable athletes to achieve greater consistency and accuracy under competition conditions.

Unlike previous studies that have primarily relied on indoor simulations, the present study was conducted on a standard outdoor archery range, offering valid insights into actual competition conditions. While these findings highlight the role of postural stability in archery performance, several limitations should be acknowledged. First, the relatively small sample size and the absence of elite athletes among the participants may limit the generalizability of the results. Second, although efforts were made to standardize weather conditions, environmental factors such as wind were not systematically controlled, and the data were collected under specific conditions that may not fully capture all competitive scenarios. Third, although biomechanical parameters such as COP displacement and limb stability were analyzed, other potentially influential factors (e.g., psychological stress or fatigue) were not considered. To address these limitations, future studies should recruit larger sample sizes, include elite archers, and employ more comprehensive measurements, potentially under both outdoor and standardized indoor conditions, to improve the precision and generalizability of the findings. In addition, future research incorporating electromyography and motion analysis is recommended to better identify the key determinants of shooting accuracy and to inform the development of effective interventions aimed at optimizing postural control and muscular coordination in archery.

## 5. Conclusions

This study examined the relationship between postural stability and shooting accuracy among competitive recurve and compound archers under standard outdoor conditions. The findings demonstrate that postural stability plays a critical role in archery performance, with recurve archers relying more on stability after arrow release, whereas compound archers depend more on stability before arrow release. These differences highlight the distinct biomechanical demands of recurve and compound archery and underscore the need for tailored training strategies. Following general physical conditioning, individualized upper-limb programs may be designed to enhance distal stability according to bow characteristics. Moreover, this study provides practical implications for coaches and athletes, suggesting that stability-focused training, biomechanical monitoring, and sport-specific conditioning may enhance both performance and injury prevention. Taken together, these results not only fulfill the objectives of the present research but also provide a foundation for future investigations aimed at optimizing archery performance.

## Figures and Tables

**Table 1:** Participant characteristics.

			Recurve Archers	Compound Archers	*p* Value	Effect Size
			Mean ± SD	Mean ± SD		
Age (years)			19.0 ± 2.5	18.7 ± 2.5	0.631	0.1
High (cm)			168.3 ± 7.4	165.7 ± 6.7	0.205	0.4
Weight (kg)			73.5 ± 14.4	65.8 ± 10.0 *	0.036	0.6
BMI (kg/m^2^)			25.9 ± 4.7	23.9 ± 3.0	0.067	0.5
Male/Female			12:7	7:11	0.140	0.4 #
Muscle strength (kgf)	Drawing side	Lower trapezius	6.4 ± 2.1	5.5 ± 1.5	0.107	0.5
		Upper trapezius	34.7 ± 8.4	32.6 ± 6.5	0.312	0.3
		Horizontal abductor	19.0 ± 4.6	15.4 ± 4.1 *	0.005	0.8
		Horizontal adductor	15.5 ± 4.0	12.6 ± 3.7 *	0.010	0.8
	Bow side	Lower trapezius	6.5 ± 2.5	5.1 ± 1.7 *	0.018	0.7
		Upper trapezius	35.0 ± 9.3	33.9 ± 6.8	0.645	0.1
		Horizontal abductor	19.1 ± 4.5	15.4 ± 4.4 *	0.005	0.8
		Horizontal adductor	15.1 ± 3.8	13.8 ± 3.7	0.221	0.3
Average scores of every arrow			8.3 ± 0.7	9.1 ± 0.3 *	0.001	1.5

* *p* < 0.05 between recurve and compound archery. # Using odds ratios.

**Table 2:** Postural stability test data.

			Recurve Archers	Compound Archers	*p* Value	Effect Size
			Mean ± SD	Mean ± SD		
Center of pressure (mm)	0.5 s before the arrow release	COPd	51.9 ± 12.7	61.6 ± 16.4 *	0.022	0.7
		COPx	1.1 ± 0.9	1.5 ± 0.6 *	0.043	0.5
		COPy	3.3 ± 2.4	3.0 ± 1.3	0.628	0.2
	0.1 s after the arrow release	COPd	13.5 ± 3.8	14.0 ± 3.8	0.629	0.1
		COPx	1.5 ± 0.9	1.5 ± 0.6	0.922	0.0
		COPy	5.4 ± 2.2	3.2 ± 0.9 *	0.001	1.3
Angular velocity (°/s)	0.5 s before the arrow release	Left ankle	1.8 ± 1.1	1.7 ± 0.3	0.716	0.1
		Wrist of bow side	12.4 ± 26.5	3.5 ± 1.6	0.115	0.5
		Wrist of drawing side	7.5 ± 8.5	3.1 ± 1.0 *	0.018	0.7
		Lower back	4.1 ± 2.3	3.4 ± 1.3	0.194	0.4
	0.1 s after the arrow release	Left ankle	3.9 ± 2.5	2.2 ± 0.4 *	0.003	0.9
		Wrist of bow side	132.6 ± 52.7	104.7 ± 36.0 *	0.037	0.6
		Wrist of drawing side	271.4 ± 73.4	215.5 ± 61.0 *	0.006	0.8
		Lower back	29.3 ± 22.1	19.8 ± 18.1	0.107	0.5

COP: center of pressure; COPd: total displacement; COPx: max medial/lateral displacement; COPy: max anterior/posterior displacement. * *p* < 0.05 between recurve and compound archery.

**Table 3:** Linear regression results of biomechanical data for predicting scores in recurve archery.

			Unstandardized Coefficients		Standardized Coefficients	95% CI	
			B	Standard Error	β	Lower	Upper
Intercept			14.502	2.502		9.997	19.006
Center of pressure	0.1 s after the arrow release	COPd	−0.109	0.047	−0.351 *	−0.202	−0.016
		COPx	−0.010	0.188	−0.007	−0.382	0.362
		COPy	0.093	0.072	0.174	−0.049	0.235
Angular velocity	0.5 s before the arrow release	Wrist of bow side	−0.002	0.008	−0.049	−0.019	0.014
		Wrist of drawing side	−0.003	0.015	−0.025	−0.032	0.027
		Lower back	0.013	0.071	0.024	−0.128	0.154
	0.1 s after the arrow release	Left ankle	−0.071	0.076	−0.143	−0.221	0.079
		Wrist of bow side	−0.007	0.003	−0.259 *	−0.012	−0.001
		Wrist of drawing side	−0.003	0.002	−0.155	−0.008	0.002
		Lower back	0.004	0.006	0.073	−0.008	0.016

Adjusted for sex, age, weight, and muscle strength. COP: center of pressure; COPd: total displacement; COPx: max medial/lateral displacement; COPy: max anterior/posterior displacement. * *p* < 0.05.

**Table 4:** Linear regression results of biomechanical data for predicting scores in compound archery.

			Unstandardized Coefficients		Standardized Coefficients	95% CI	
			B	Standard Error	β	Lower	Upper
Intercept			11.847	1.448		8.979	14.714
Center of pressure	0.5 s before the arrow release	COPx	−0.169	0.122	−0.170	−0.411	0.073
		COPy	−0.121	0.058	−0.242 *	−0.235	−0.007
	0.1 s after the arrow release	COPd	−0.022	0.024	−0.121	−0.069	0.024
		COPx	0.083	0.119	0.070	−0.153	0.319
		COPy	0.115	0.073	0.168	−0.028	0.259
Angular velocity	0.5 s before the arrow release	Left ankle	−0.058	0.241	−0.025	−0.535	0.420
		Wrist of bow side	−0.061	0.054	−0.135	−0.168	0.047
		Wrist of drawing side	−0.191	0.080	−0.259 *	−0.349	−0.034
		Lower back	0.150	0.082	0.237	−0.012	0.312
	0.1 s after the arrow release	Left ankle	0.097	0.196	0.059	−0.292	0.486
		Wrist of bow side	−0.001	0.002	−0.035	−0.006	0.004
		Wrist of drawing side	−0.001	0.001	−0.096	−0.004	0.001
		Lower back	−0.004	0.006	−0.090	−0.016	0.007

Adjusted for sex, age, weight, and muscle strength. COP: center of pressure; COPd: total displacement; COPx: max medial/lateral displacement; COPy: max anterior/posterior displacement. * *p* < 0.05.

## Data Availability

The data that support the findings of this study are not publicly available due to privacy and ethical restrictions but are available from the corresponding author upon reasonable request.

## Supplementary Materials

The following supporting information can be downloaded at https://www.mdpi.com/article/10.3390/sports13090310/s1: Supplementary Table S1. Correlation coefficients of scores versus biomechanical data for recurve archery. Supplementary Table S2. Correlation coefficients of scores versus biomechanical data for compound archery.
