# Supplementary material for: Associations of Scoring Accuracy with Postural Stability and Strength Measures in Archers on a Standard Archery Site
Source: Sports (Basel). 2025 Sep 8;13(9):310. doi: 10.3390/sports13090310 (PMC12473916; doi:10.3390/sports13090310)
Supplement: Supplementary file 1 [file sports-13-00310-s001.zip › Supplementary Table S1.pdf]

Supplementary Table S1. Correlation coefficients of scores versus biomechanical data for recurve archery.

				Center of pressure						Angular velocity							
				0.5s before the arrows were thrown			0.1s before the arrows were thrown			0.5s before the arrows were thrown				0.1s before the arrows were thrown			
			Scores	COPd	COP x	COPy	COPd	COP x	COPy	Left ankle	Wrist of bow side	Wrist of drawing side	Lower back	Left ankle	Wrist of bow side	Wrist of drawing side	Lower back
		Scores	1														
COP	0.5s before the arrows were thrown	COPd	-.137	1													
		COP x	-.051	.380**	1												
		COPy	-.070	.254**	.860**	1											
	0.1s before the arrows were thrown	COPd	-.192*	.839**	.354**	.238**	1										
		COP x	-.048	.373**	.666**	.410**	.484**	1									
		COPy	-.125	.353**	.358**	.313**	.690**	.430**	1								
Angular velocity	0.5s before the arrows were thrown	Left ankle	-.082	.272**	.820**	.849**	.223**	.531**	.278**	1							
		Wrist of bow side	-.022	.228**	.773**	.680**	.245**	.485**	.390**	.705**	1						
		Wrist of drawing side	-.122	.310**	.731**	.804**	.269**	.406**	.236**	.930**	.520**	1					
		Lower back	-.121	.291**	.573**	.630**	.273**	.150	.333**	.664**	.563**	.595**	1				
	0.1s before the arrows were thrown	Left ankle	-.178*	.370**	.662**	.659**	.452**	.473**	.502**	.715**	.709**	.665**	.650**	1			
		Wrist of bow side	-.210**	.072	-.175*	-.164*	.216**	-.172*	.305**	-.179*	-.166*	-.114	.041	.062	1		
		Wrist of drawing side	-.151	.126	-.113	-.036	.105	-.070	.115	-.086	-.289**	.043	-.064	-.029	.480**	1	
		Lower back	-.057	.187*	-.069	-.075	.298**	-.135	.305**	-.104	-.103	-.030	.258**	.195*	.351**	.349**	1

COP: center of pressure; COPd: total center of pressure displacement; COPx: maximum amplitude of center of pressure displacement in the medial/lateral direction; COPy: maximum amplitude of center of pressure displacement in the anterior/posterior direction.

$^{*}p < 0.05$; $^{**}p < 0.01$.
